# MicroRNA-590 Inhibits Lipoprotein Lipase Expression and Prevents Atherosclerosis in apoE Knockout Mice

**DOI:** 10.1371/journal.pone.0138788

**Published:** 2015-09-23

**Authors:** Ping-Ping He, Xin-Ping OuYang, Yuan Li, Yun-Cheng Lv, Zong-Bao Wang, Feng Yao, Wei Xie, Yu-Lin Tan, Liang Li, Min Zhang, Gang Lan, Duo Gong, Hai-Peng Cheng, Hui-Juan Zhong, Dan Liu, Chong Huang, Zhao-Xia Li, Xi-Long Zheng, Wei-Dong Yin, Chao-Ke Tang

**Affiliations:** 1 Institute of Cardiovascular Research, Key Laboratory for Atherosclerologyof Hunan Province,Hunan Province Cooperative innovation Center for Molecular Target New Drug Study, University of South China,Hengyang, Hunan 421001, China; 2 Hunan Province Cooperative innovation Center for Molecular Target New Drug Study, 28 West Changsheng Road, Hengyang 421001, Hunan, China; 3 Nursing School, University of South China, Hengyang 421001, Hunan, China; 4 Department of Physiology, The Neuroscience Institute, Medical College, University of South China,Hengyang, Hunan, 421001, China; 5 Pharmacy and Biological Science College, University of South China, Hengyang, Hunan, 421001, China; 6 Department of Cardiovascular Medicine, The Liwan Hospital of Guangzhou Medical University, Guangzhou, Guangdong, 510170, China; 7 Department of Biochemistry and Molecular Biology, The Libin Cardiovascular Institute of Alberta, Cumming School of Medicine, The University of Calgary, Health Sciences Center, 3330 Hospital Dr NW, Calgary, Alberta, Canada; University of Kansas Medical Center, UNITED STATES

## Abstract

Recent studies have suggested that miR-590 may play critical roles in cardiovascular disease. This study was designed to determine the effects of miR-590 on lipoprotein lipase (LPL) expression and development of atherosclerosis in apolipoprotein E knockout (apoE^−/−^) mice and explore the potential mechanisms. En face analysis of the whole aorta revealed that miR-590 significantly decreased aortic atherosclerotic plaque size and lipid content in apoE^−/−^ mice. Double immunofluorescence staining in cross-sections of the proximal aorta showed that miR-590 agomir reduced CD68 and LPL expression in macrophages in atherosclerotic lesions. MiR-590 agomir down-regulated LPL mRNA and protein expression as analyzed by RT-qPCR and western blotting analyses, respectively. Consistently, miR-590 decreased the expression of CD36 and scavenger receptor A1 (SRA1) mRNA and protein. High-performance liquid chromatography (HPLC)analysis confirmed that treatment with miR-590 agomir reduced lipid levels either in plasma orinabdominal cavity macrophages of apoE^−/−^ mice. ELISA analysis showed that miR-590 agomir decreased plasma levels of pro-inflammatory cytokines, such as tumor necrosis factor-alpha (TNF-α), monocyte chemotactic protein-1 (MCP-1), interleukin-1β (IL-1β)and interleukin-6 (IL-6). In contrast, treatment with miR-590 antagomir prevented or reversed these effects. Taken together, these results reveal a novel mechanism of miR-590 effects, and may provide new insights into the development of strategies for attenuating lipid accumulation and pro-inflammatory cytokine secretion.

## Introduction

Cardiovascular disease caused by atherosclerosis is the number one cause of death in Western countries and threatens to become the major cause of morbidity and mortality worldwide[1]. Nowadays the incidence of cardiovascular disease is going up rapidly in epidemic proportions in developing countries[2].Atherosclerotic lesions are characterized by the accumulation of abundant lipids and macrophage-derived foam cells. The later results from the cellular uptake of modified low-density lipoprotein (LDL) and triglyceride-rich lipoproteins (TRL), such as very low-density lipoprotein (VLDL) remnants, after infiltration of macrophages into the arterial wall [3, 4]. Inflammatory mechanisms are also critical in the progression of each characteristic lesion/stage of atherogenesis and the associated thrombotic complications[5]. Evidence for a link between lipid-loading and amplification of inflammatory response suggests that it is important to seek for some strategies to reduce lipid accumulation and inflammation response so as to prevent and treat atherosclerotic diseases.

Lipoprotein lipase (LPL) is an important enzyme that hydrolyzes triglyceride (TG) in lipoproteins, especially in TRL such as chylomicrons (CM) and VLDL, to liberate fatty acid (FA). The relationship between LPL and atherosclerosis has been controversial for long time. LPL is generally viewed as an anti-atherogenic enzyme, but none of its atherogenic characters is known to involve macrophage LPL. Macrophage LPL is thought to act as a molecular bridge between proteoglycans and lipoprotein receptors, such as remnant-like particles and LDL receptors, to induce the retention and uptake of atherogenic lipoproteins[6]. In addition, macrophage LPL stimulates the secretion of inflammatory cytokines and promotes the formation of atherosclerotic lesions. It has been demonstrated that expression of LPL by the macrophage promoted foam cell formation and development of atherosclerosis [7].Kawashima revealed that suppression of LPL expression was correlated with up-regulation of ABCA1 mRNA levels, and resulted in an apparent increase in subsequent ABCA1-dependent cholesterol efflux [8]. Takahashi et al. also showed that intracellular triglyceride levels and CD36 and carnitine palmitoyltransferase-1 mRNA levels were reduced in MLpLKO/ApoEKO macrophages compared with ApoEKO macrophages[9]. Indeed, macrophage LPL plays an important role in the development of atherosclerosis because of its promoting effects onpro-inflammatory cytokine expression and lipid composition.

The discovery of microRNAs (miRNAs), small noncoding RNA molecules involved in the posttranscriptional regulation of gene expression, has greatly influenced and furthered our understanding of the pathogenesis of atherosclerosis[10, 11].Recent studies have demonstrated that miR-590, a small miRNA located on the proximal end of the long arm of human genome chromosome 7, may have critical roles in cardiovascular disease, such asvasoprotection, regulation of lipid metabolism, prevention of myocardial infarction and cardiac regeneration, through regulating its target genes[12–14]. Additionally, down-regulation of miR-590 expression has been found in cardiovascular disease[15]. However, how miR-590 affects the development of atherosclerosis has not yet been fully elucidated.

We previously demonstrated that miR-590 can attenuate lipid accumulation and pro-inflammatory cytokine secretion by targeting LPL gene in human THP-1 macrophages[16]. Thus, in this study, we further investigated the possible effects of miR-590 on LPL expression and atherosclerotic lesions *in vivo*. Our results revealed that miR-590 inhibited LPL expression and prevented atherosclerotic lesions in apoE^−/−^ mice.

## Materials and Methods

### Animal Models and Treatments

ApoE^−/−^ male mice aged 8-weeks were purchased from Nanjing CAVENS Biological Technology Co, Ltd. ApoE^−/−^ mice were randomized into four groups: miR-590 agomir group (AG), miR-590 scrambled agomir negative control group (AG-NC), miR-590 antagomir group (AN) and miR-590 scrambled antagomir negative control group (AN-NC).There were 15 mice in each group. All mice were maintained on a 12 h light/dark cycle with unlimited access to food and water. Mice were fed the high fat/high cholesterol Western diet (15% fat wt/wt, 0.25% cholesterol wt/wt) and injected via tail vein with miRNA agomir/antagomir or their respective controls (GuangZhou RiboBio. Co.) at a dose of 80mg /kg wt in 0.2 ml saline once every four weeks. After 8 weeks, these animals were anesthetized (3% isoflurane) andeuthanized by exsanguination. Hearts, aortas, abdominal cavity macrophages and blood samples were collected for the measurements as indicated in other parts. The animal study was performed under the Guide for the Care and Use of Laboratory Animals published by the US National Institutes of Health (NIH publication no. 85–23, revised in 1996) and Care and Use guidelines for experimental animals of University of South China. The study protocol was approved by the Animal Ethics Committee of University of South China.

### Assessment of Atherosclerosis

Hearts and proximal aortas were removed and fixed with formalin[17]. The hearts were oriented in the direction where the three valves of aortic root were in the same plane. 8μm sections were cut and placed on the glass slides. To evaluate atherosclerotic lesions at aortic root, hearts from mice were stained with hematoxylin and eosin (H&E) stains. To examine lipid accumulation in atherosclerotic lesions, mouse hearts were stained with Oil red O. To quantify collagen fibers in atherosclerotic lesions, mouse hearts were stained with Masson’s trichrome staining. Lesion area was quantified in every fourth section, and the average was reported from five measurements. These samples were photographed using a Nikon 80I Eclipse equipped with Nikon DS-EI1 camera. Lesion areas were quantified using IMAGEPRO PLUS software.

### Immunofluorescence Assay

Tissues collected for morphological analysis with aortic roots and ascending aorta were prepared for OCT-embedded cryosections. The sections were fixed in 4% parafor maldehyde for 30 min, washed in PBS, and then incubated in buffered normal goat serum to prevent non-specific binding of antibodies for 1 h at room temperature. The sections were then incubated separately overnight with antibodies against CD68 or LPL (1:100; Santa Cruz Biotechnology, CA, USA), followed by incubation with Alexa Fluor 592-conjugatged goat anti-rabbit IgG (1:200;Invitrogen, Carlsbad, CA, USA) for 1 h at 37°C in a humidified box. Thereafter, the sections were washed in PBS and counter-stained with Hoechst dyetostain DNA and illuminate the nuclei. Photomicrographs were taken at random using an Olympus BX-URA2 camera in 4 sections per mouse sample.

### Western Blot Analysis

Aortic tissues and macrophages in abdominal cavities were lysed for extraction of protein using RIPA buffer containing proteinase inhibitor cocktails (Sigma) as previously described[18]. Protein expression of LPL was examined using western blotting. In brief, 20 μg of protein lysates from isolated aortic tissues or peritoneal macrophages were loadedin each lane. The resulting blots were probed with primary antibodies against LPL or β-actin (1:1000, Sigma), followed by incubation with the appropriate secondary antibodies (1:2000, Sigma). The proteins were visualized using a chemiluminescence method and quantified by densitometry.

### Quantitative Real-Time PCR Analysis

Aortic tissues and abdominal cavity macrophages were lysed for RNA extraction using the RNeasy Mini Kit (QIAGEN)[19]. LPL mRNA expression was examined using RT-qPCR. The sequences of the real time PCR primers from Biology Engineering Corporation in Shanghai of China were: LPL, forward: 5’-GGGAGTTTGGCTCCAGAGTTT-3’, and reverse: 5’-TGTGTCTTCAGGGGTCCTTAG-3’.β-actin was used as the internal control.

### Lipid Analyses

Mice were fasted overnight and then sacrificed. Blood samples were obtained from the retro-orbital plexus. Triglyceride (TG), total cholesterol (TC), and high-density lipoprotein cholesterol (HDL-C) were determined by commercially enzymatic methods (test kits, Nanjing Jiancheng Biotech Inc. Shanghai, China), according to the manufacturer’s instructions. The sterol analyses were performed using high-performance liquid chromatography (HPLC) analysis (Model 2790, controlled with Empower Pro software; Waters Corp., Milford, MA)[20].

### Cytokine ELISA

The quantitation of secreted pro-inflammatory cytokines was performed by enzyme-linked immunosorbent assay (ELISA). Tumor necrosis factor-alpha (TNF-α), monocyte chemotactic protein-1 (MCP-1), interleukin-1β (IL-1β) and IL-6 in serum were analyzed according to the manufacturer’s instructions[21]. Quantitative determinations in three different experiments were performed.

### Statistical Analysis

All results were expressed as mean ± standard deviation (SD). Statistically significant differences among groups were analyzed by one-way analysis of variance (ANOVA) or Student’s t-test using SPSS 18.0 software. Statistical significance was indicated when *p* values were less than 0.05.

## Results

### MiR-590 prevents progression of aortic atherosclerosis in apoE^−/−^ mice

**Fig 1:**
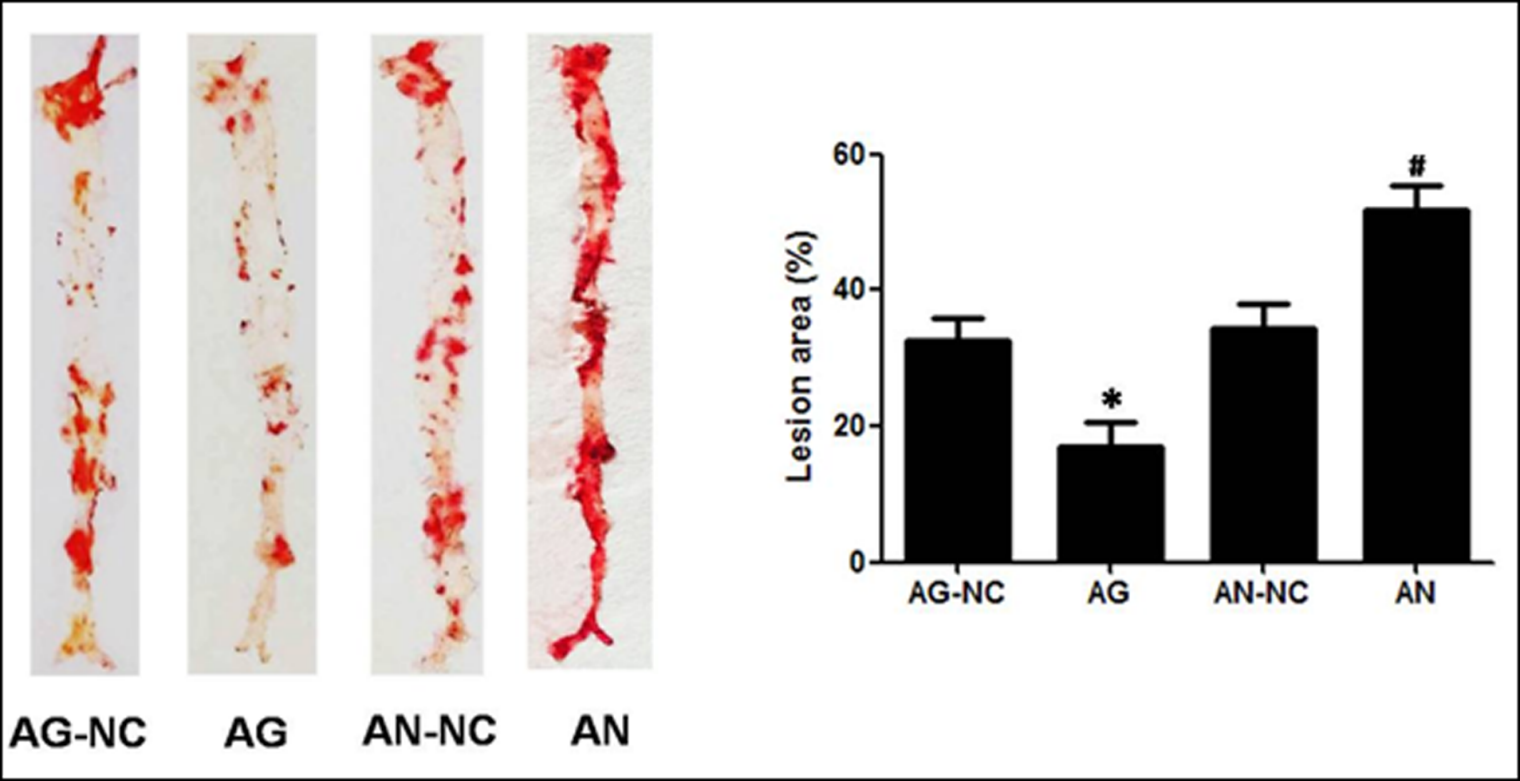
Effects of miR-590 on atherosclerotic lesion areas in apoE^−/−^ mice. Representative images and the quantification of atherosclerotic lesion areas in the en faceanalysis of the wholeaorta with Sudan IV staining were obtained from apoE^-/-^ mice fed Western diet. Male 8-week-old apoE^-/-^ mice were given a tail vein injection with miR-590 agomir (AG) or its scrambled agomir negative control (AG-NC), miR-590 antagomir (AN) or its scrambled antagomir negative control (AN-NC). Total Sudan IV staining positive area was determined using IMAGEPRO PLUS Software. Values are mean ± SD.*: P<0.05 *vs*AG-NC. #: P<0.05 *vs* AN-NC.

**Fig 2:**
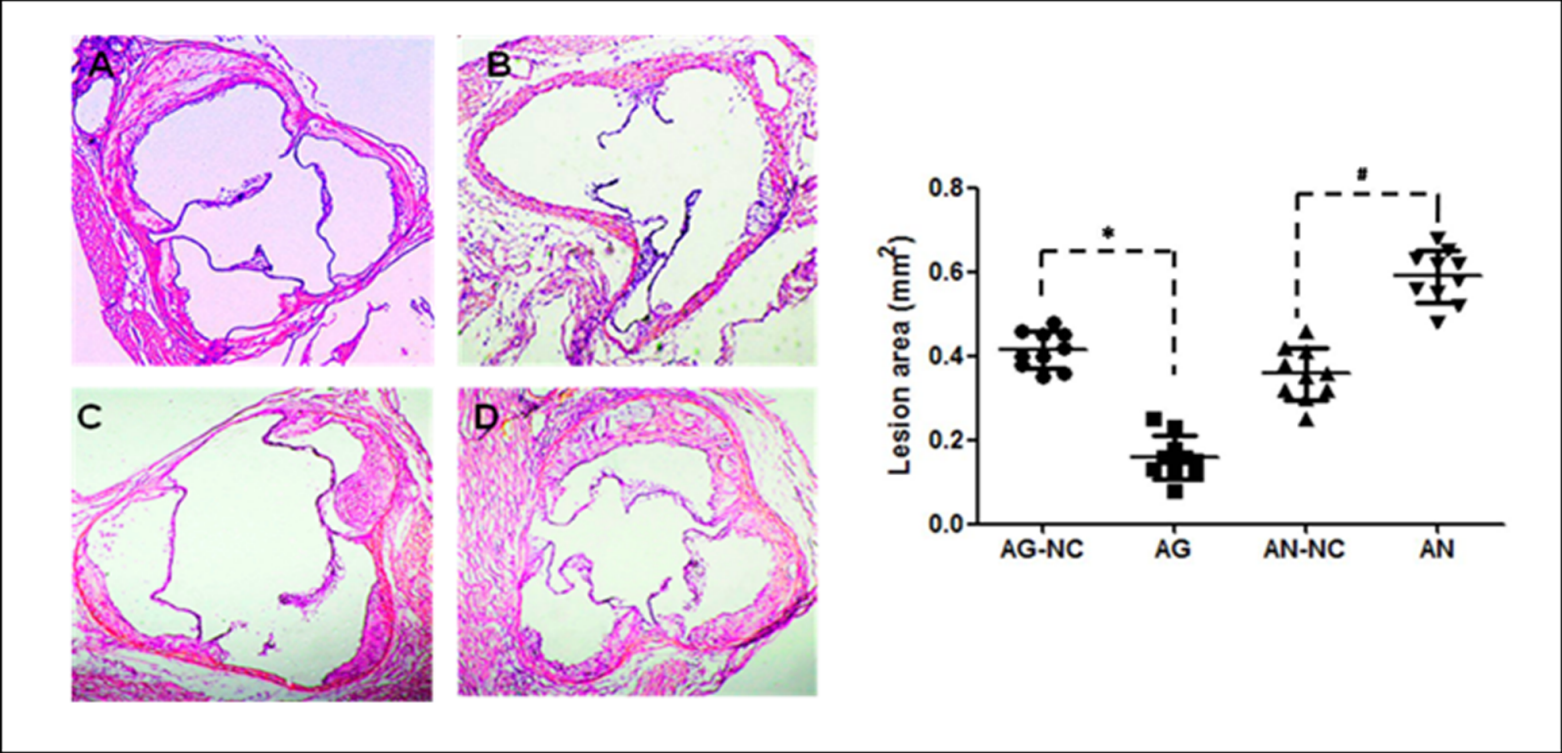
Effects of miR-590 on aortic atherosclerotic lesions in aortic root in apoE^−/−^ mice. Representative micrographs were obtained from hematoxylin-eosin staining of cross-sections of proximal aorta in apoE^-/-^ mice. Atherosclerotic lesions were evaluated in mice injected with miR-590 agomir negative control (AG-NC, A), miR-590 agomir (AG, B), miR-590 antagomir negative control (AN-NC, C) and miR-590 antagomir (AN, D). Values are mean ±SD.*: P<0.05 *vs* AG-NC. #: P<0.05 *vs* AN-NC.

**Fig 3:**
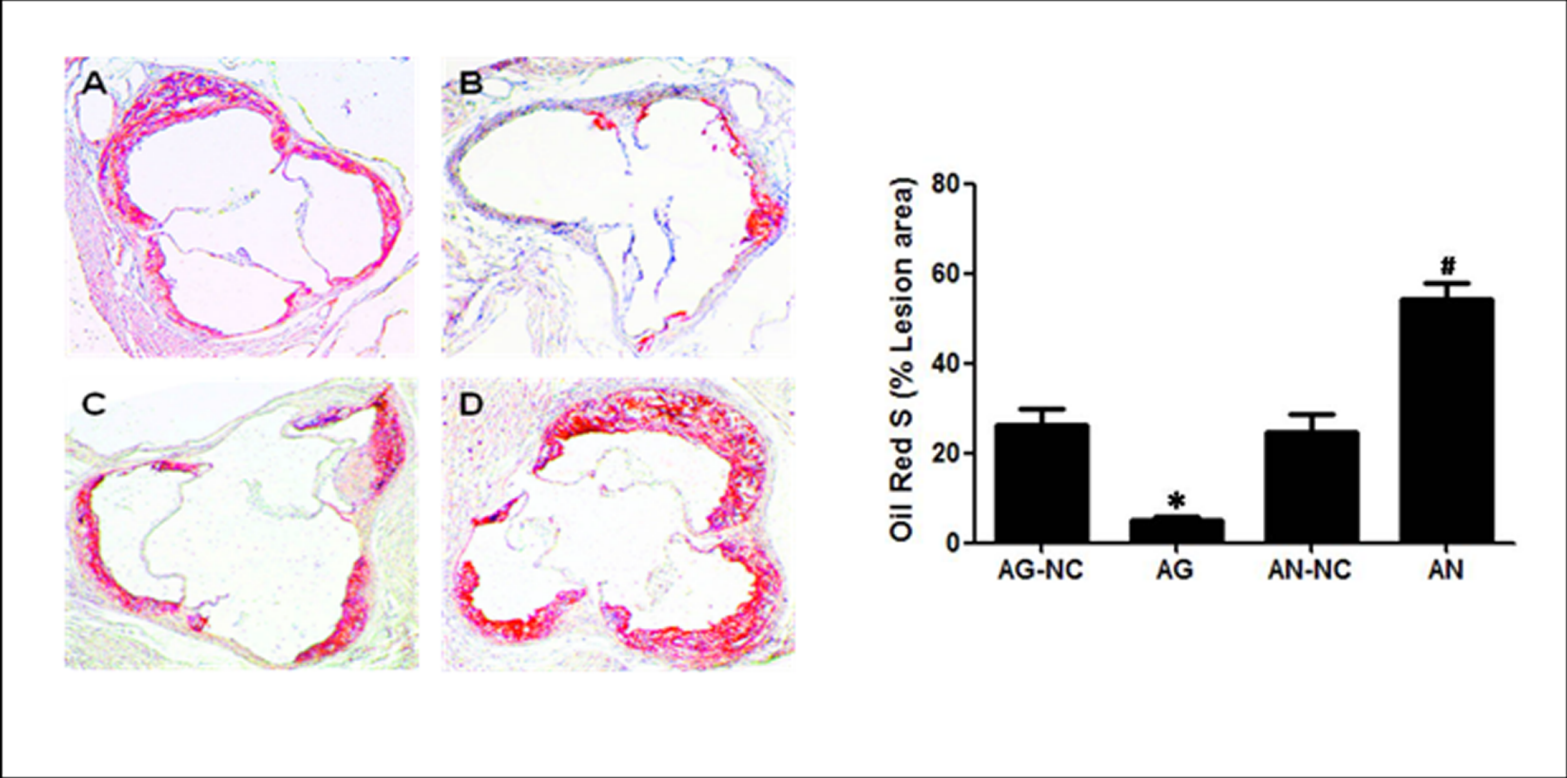
Effects of miR-590 on the aortic sinus lesion areas in apoE^−/−^ mice. Characterization of aortic sinus atherosclerotic lesion areas was performed by Oil red O stainingin mice treated with miR-590 agomir negative control (AG-NC, A), miR-590 agomir (AG, B), miR-590 antagomir negative control (AN-NC, C) and miR-590 antagomir (AN, D). Images of representative sections from each group are accompanied by summarized bar charts. Original magnification: 10×4. Total Oil red O staining positive area was determined using IMAGEPRO PLUS Software. Values are mean ±SD.*: P<0.05 *vs* AG-NC. #: P<0.05 *vs* AN-NC.

It is known that atherosclerosis is characterized by the progressive pathological changes, and the rupture of an advanced atherosclerotic lesion may cause the death of patients with atherosclerotic coronary artery disease[22]. In this study, we used miR-590 gain-of-function and miR-590 loss-of-function approaches by taking advantage of miR-590 agomir and miR-590 antagomir to examine whether miR-590 overexpression and inhibition influenced the formation of atherosclerotic plaque in apoE^−/−^ mice fed high fat diet. In each experimental group, no significant adverse reaction was observed. The atherosclerotic lesion area was examined by an en face analysis of the whole aorta and the cross-sections of the proximal aorta. The en face analysis of the whole aorta showed that atherosclerotic lesions throughout the aorta in apoE^−/−^ mice were significantly decreased in miR-590 agomir-treated group, but increased in miR-590 antagomir-treated group when compared with their respective negative control mice (Fig 1). The plaque area in the proximal aorta was extensively examined in the hematoxylin and eosin-stained cross-sections of the aortic root. We observed that the plaque area in miR-590 agomir-treated mice was less than that in mice treated with miR-590 scrambled agomir. On the other hand, plaques in miR-590 antagomir group were more severe than those in miR-590 scrambled antagomir group (Fig 2). In addition, severity of lipid deposition in proximal aorta and the stained portions of cross-sections was reduced by treatment with miR-590 agomir, but enhanced by treatment with miR-590 antagomir, compared with their respective control groups (Fig 3). Taken together, these findings clearly indicate that miR-590 inhibits the development and progression of atherosclerotic lesions.

### MiR-590 reduces the expression of macrophage LPL in atherosclerotic lesions of apoE^−/−^ mice

**Fig 4:**
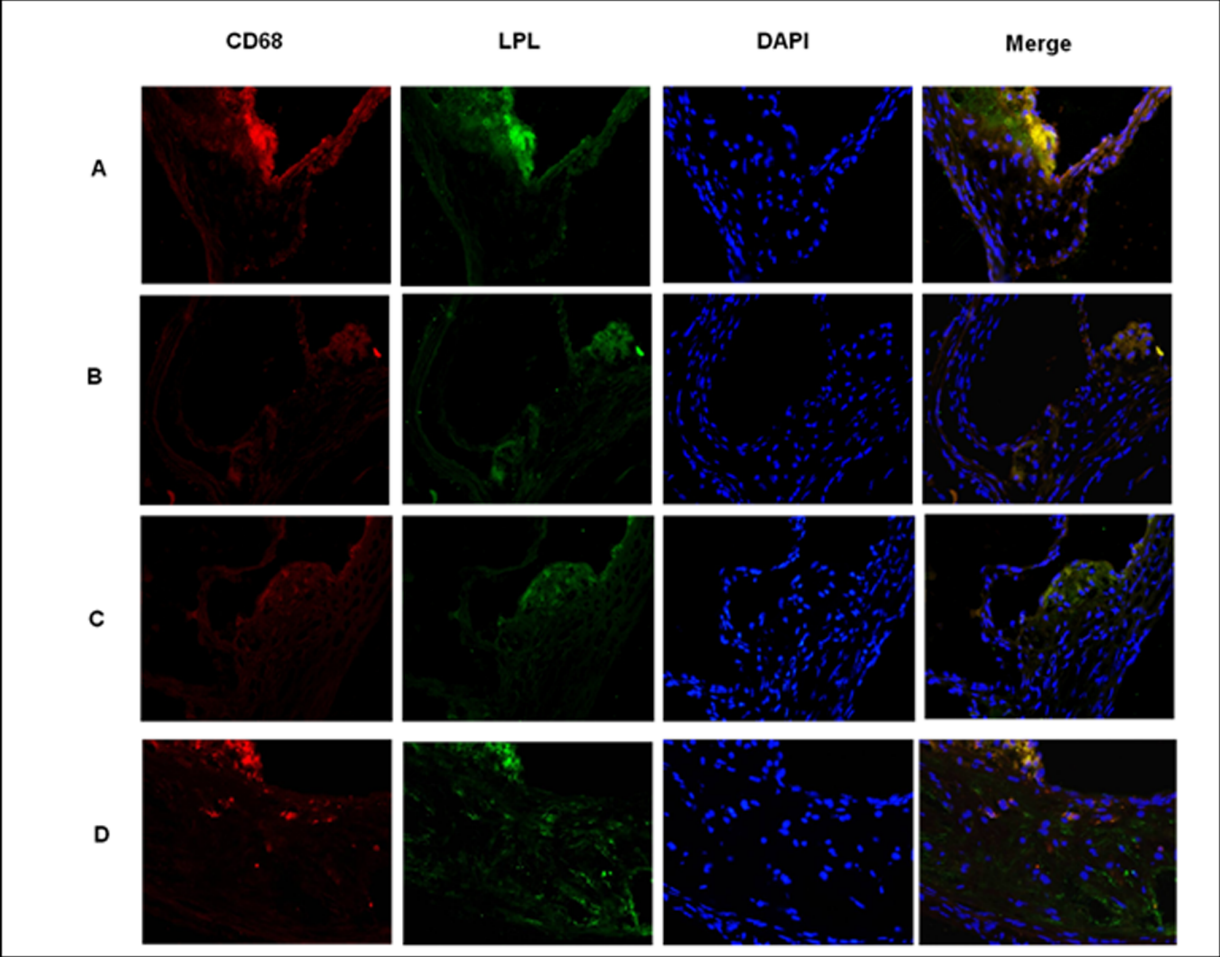
Expression of LPL in macrophages in the aortic roots of apoE^−/−^ mice. Representative images show the localization of LPL (green), CD68 (red) and DAPI (blue) staining in aortic tissues derived from mice treated with miR-590 agomir negative control (AG-NC, A), miR-590 agomir (AG, B), miR-590 antagomir negative control (AN-NC, C) and miR-590 antagomir (AN, D). Original magnification: 10×40. Frozen sections of the aortic roots were stained with anti-LPL and anti-CD68 antibodies, followed by observation under a fluorescence microscope.

We then tested whether miR-590 affected the expression of macrophage LPL in atherosclerotic lesions in the aortic sinus of apoE^−/−^ mice. To do so, we used double immunofluorescence assay to determine the correlation of LPL and macrophages in atherosclerotic lesions in the cross-sections of the proximal aorta. Notably, our results showed that LPL-positive staining area was overlapped with macrophage-rich area in aortic atherosclerotic lesion. Furthermore, mice treated with miR-590 agomir had obvious less staining for macrophage LPL in atherosclerotic lesions than mice treated with miR-590 scrambled agomir. However, mice in miR-590 antagomir-treated group displayed an increase in macrophage LPL stainingin comparison with its control group (Fig 4). These data suggest an inhibitory effect of miR-590 on macrophage LPL in atherosclerotic lesions.

### MiR-590 inhibits LPL mRNA and protein expression in apoE^−/−^ mice

**Fig 5:**
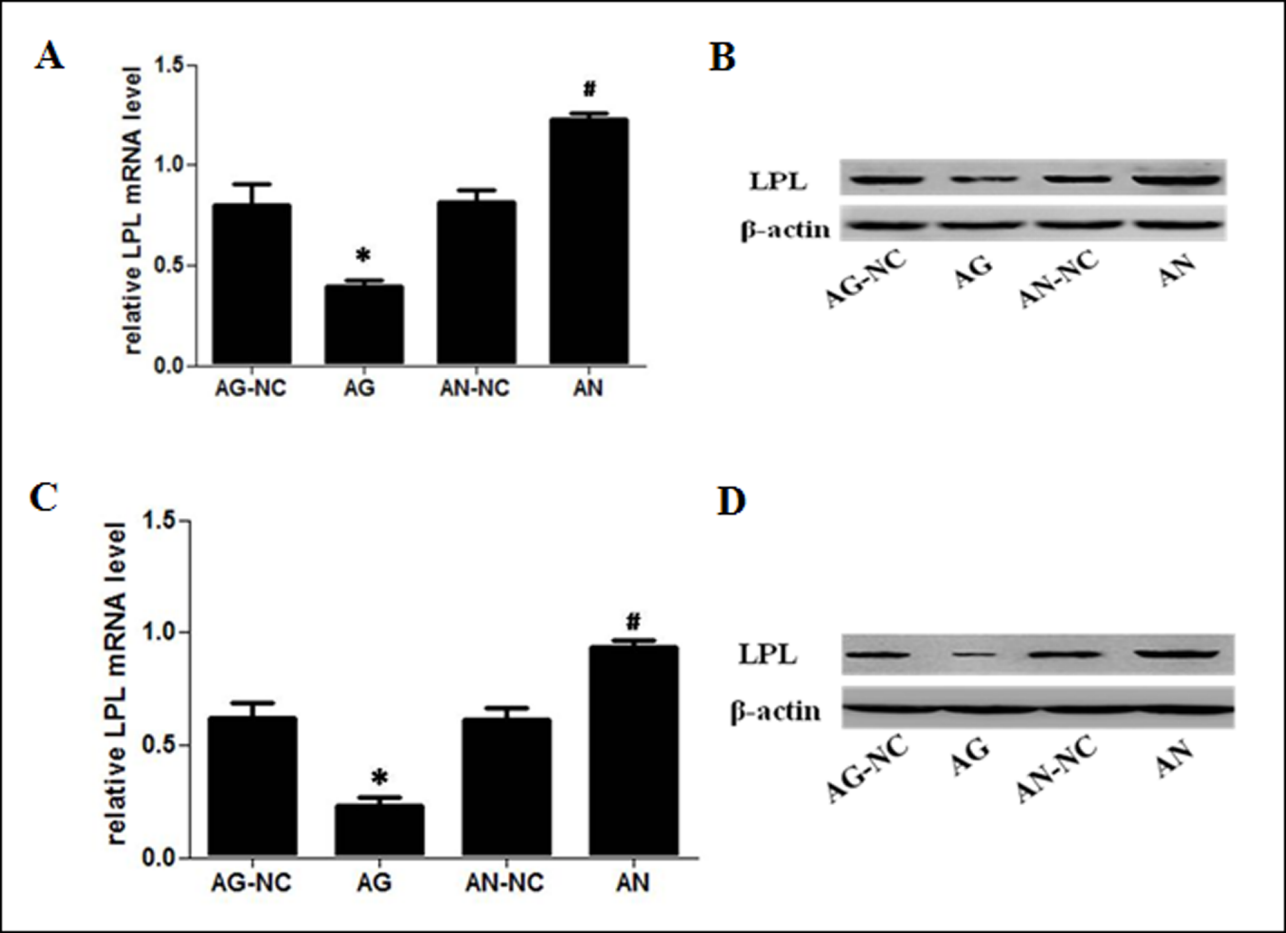
Effects of miR-590 on LPL expression in apoE^−/−^ mice. (A) LPL mRNA levels in aortic tissues of apoE^−/−^ mice as measured by RT-qPCR. (B) LPL protein levels in aortic tissues of apoE^−/−^ mice as measured by western blotting. (C) LPL mRNA levels in peritoneal macrophages of apoE^−/−^ mice as measured by RT-qPCR. (D) LPL protein levels in peritoneal macrophages of apoE^−/−^ mice as measured by western blotting. Values are mean ± SD.*: P<0.05 *vs* AG-NC. #: P<0.05 *vs* AN-NC.

Using bioinformatic analyses and dual-luciferase reporter assays, we found that miR-590 directly inhibited LPL expression by targeting LPL 3’UTR *in vitro*[16]. Therefore, we further investigated the effects of miR-590 on LPL expression *in vivo*. The aortic arch from each group was homogenized for the determination of LPL levels using RT-qPCR and western blot analyses. Our data showed that the expression levels of LPL mRNA and protein in aortic roots in apoE^−/−^mice were significantly reduced in miR-590 agomir-treated group, but increased in miR-590 antagomir-treated group, compared with their respective control groups (Fig 5A and 5B).

Meanwhile, miR-590 agomir treatment significantly reduced the expression levels of LPL mRNA and protein in peritoneal macrophages of apoE^−/−^ mice, but miR-590 antagomir treatment increased LPL mRNA and protein expression, compared with their respective control groups (Fig 5C and 5D). These data illustrated that miR-590 directly inhibited macrophage LPL expression in apoE^−/−^ mice.

### MiR-590 decreases plasma cholesterol levels in apoE^−/−^ mice

Considering that LPL hydrolyzes triglyceride and impacts lipid levels, we next examined potential association between miR-590 and lipid levels in atherosclerotic animal models. As shown in Table 1, apoE^−/−^ mice in miR-590 agomir-treated group had a significant increase in TG levels, but decrease in both TC and LDL-C levels in plasma compared with control mice treated with miR-590 scrambled agomir. On the other hand, miR-590 antagomir treatment decreased TG levels, but increased TC and LDL-C levels in comparison with its control group. Taken together, these findings indicated that miR-590 lowers plasma cholesterol levels in apoE^−/−^ mice.

**Table 1:** Body weight and plasma lipid profile in apoE^−/−^ mice ($\bar{X}$+S).

	AG-NC	AG	AN-NC	AN
**BW(g)**	**27.32±2.23**	**28.30±1.35**	**28.83±3.68**	**29.36±1.65**
**TG(mmol/L)**	**2.28±0.62**	**3.52±0.83** *	**2.31±0.68**	**1.35±0.29** ^**#**^
**TC(mmol/L)**	**18.68±2.21**	**15.79±1.96** *	**19.02±1.36**	**22.86±2.58** ^**#**^
**HDL-C(mmol/L)**	**3.02±2.15**	**3.10±0.23**	**3.14±1.25**	**3.12±0.63**
**LDL-C(mmol/L)**	**15.10±2.23**	**12.35±1.20** *	**15.26±1.53**	**17.42±3.05** ^**#**^

AG-NC, miR-590 scrambled agomir negative control; AG, miR-590 agomir; AN-NC, miR-590 scrambled antagomir negative control; AN, miR-590 antagomir; BW, body weight; TG, Triglyceride; TC, Total Cholesterol; HDL-C, High Density Lipoprotein Cholesterol; LDL-C, Low Density Lipoprotein Cholesterol. Plasma lipids from different experimental groups were measured with enzymatic methods. The data were the means ± SEM from the indicated numbers of male apoE^-/-^ mice in each group (n = 15).

*: P<0.05, vs AG-NC.

#: P<0.05, vs AN-NC.

### MiR-590 reduces lipid accumulation in peritoneal macrophages of apoE^−/−^ mice

LPL in macrophages is known to play a key role in cholesterol homeostasis and pro-atherosclerosis[23]. Therefore, we performed HPLC to measure cellular cholesterol levels in peritoneal macrophages of apoE^−/−^ mice in response to treatment with miR-590 agomir or miR-590 antagomir or their respective scrambled controls. As anticipated, cellular levels of TC and cholesterol ester (CE) in peritoneal macrophages in miR-590 agomir-treated group were significantly lower than those in the control group. In comparison with its control group, however, cellular cholesterol levels were increased in response to treatment with miR-590 antagomir (Table 2). These findings suggested that miR-590 reduces lipid accumulation in macrophages.

**Table 2:** Effects of miR-590 on lipid accumulation in macrophages in abdominal cavity of apoE^−/−^ mice ($\bar{X}$±S).

	AG-NC	AG	AN-NC	AN
**TC**	**512±28**	**405±27** ^*^	**520±23**	**605±15** ^**#**^
**FC**	**185±32**	**220±20** ^*^	**189±28**	**152±31** ^**#**^
**CE**	**327±30**	**185±26** ^*^	**331±24**	**453±24** ^**#**^
**CE/TC(%)**	**63.9**	**45.7**	**63.7**	**74.9**

Unit: (mg/g protein); TC: Total Cholesterol; FC: Free Cholesterol; CE: Cholesterol Ester.

*: P<0.05, vs AG-NC.

#: P<0.05, vs AN-NC.

### MiR-590 reduces the mRNA and protein expression of SRA1 and CD36 in peritoneal macrophages of apoE^−/−^ mice

**Fig 6:**
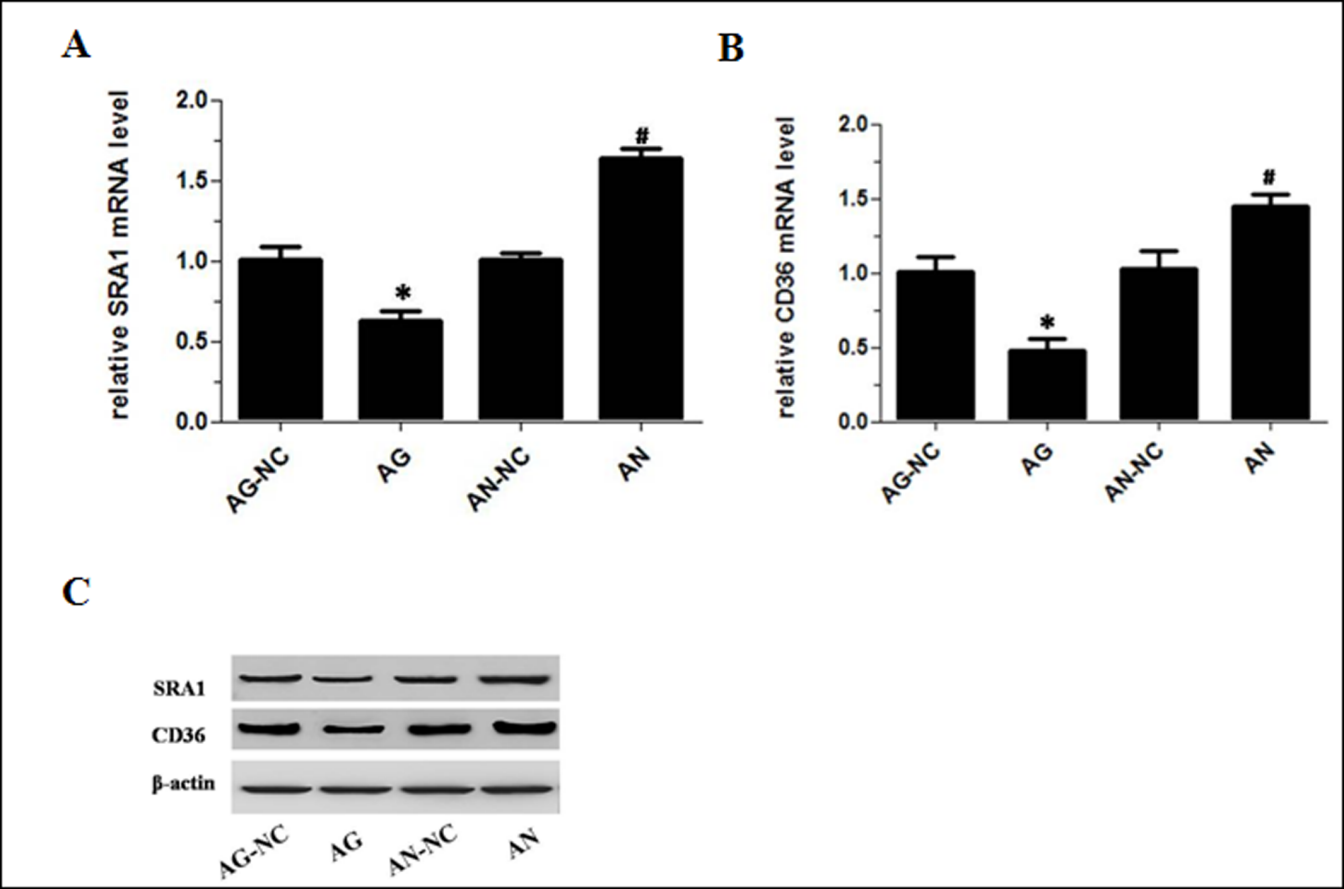
Effects of miR-590 on SRA1 and CD36 expression in apoE^−/−^ mice. (A) SRA1 mRNA levels in peritoneal macrophages of apoE^−/−^ mice as measured by RT-qPCR. (B) CD36 mRNA levels in peritoneal macrophages of apoE^−/−^ mice as measured by RT-qPCR. (C) SRA1 and CD36 protein levels in peritoneal macrophages of apoE^−/−^ mice as measured by western blotting. Values are mean ± SD.*: P<0.05 *vs* AG-NC. #: P<0.05 *vs* AN-NC.

It was previously reported that macrophage LPL could up-regulate the expression of SR-A or CD36, two principal receptors responsible for the uptake of modified LDL in macrophages[24, 25].Therefore we determined the effect of miR-590 on SRA1 and CD36 expression in macrophages from abdominal cavities of apoE^−/−^ mice. As expected, the expression of both SRA1 and CD36 mRNA and protein was reduced in mice treated with miR-590 agomir, but increased in mice treated with miR-590 antagomir (Fig 6A, 6B and 6C), when compared with their respective control group. These findings suggested that miR-590 reduces SRA1 and CD36 expression via suppressing LPL levels.

### MiR-590 reduces the secretion of plasma pro-inflammatory cytokines in apoE^−/−^ mice

Given the relationship between miR-590 and LPL, we utilized ELISA to determine whether miR-590 affected the secretion of plasma pro-inflammatory cytokines in apoE^−/−^ mice. Our results showed that the levels of TNF-α, MCP-1, IL-1β and IL-6 were significantly decreased in mice treated with miR-590 agomir compared with those in the control group. In contrast, miR-590 antagomir treatment increased TNF-α, MCP-1, IL-1β and IL-6 levels in the blood of apoE^−/−^ mice (Figs A, B, C and D in S1 File).Therefore, our findings suggested that miR-590 had an inhibitory effect on inflammation response.

## Discussion

**Fig 7:**
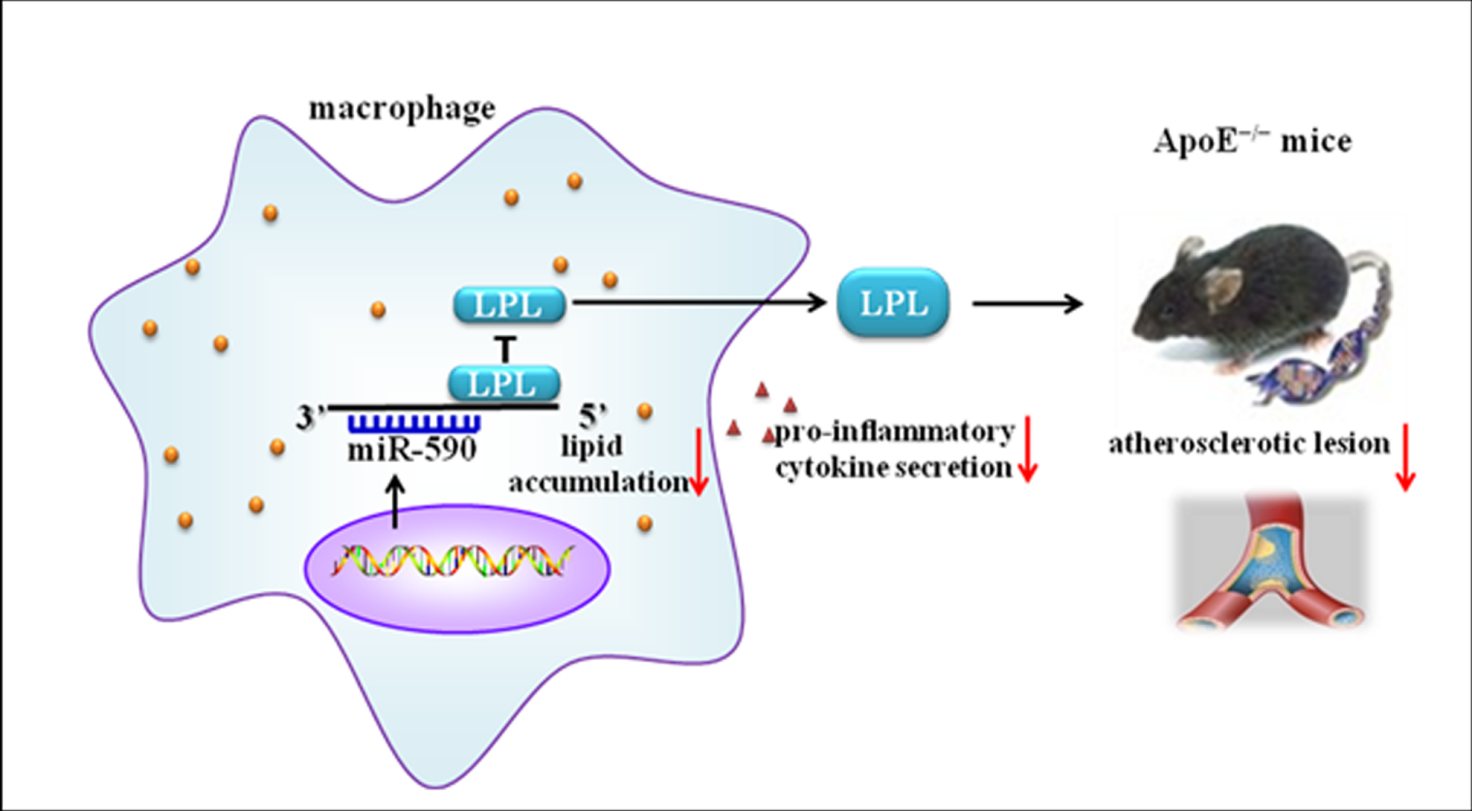
Protective effects of miR-590 on atherosclerosis in apoE^−/−^ mice. MiR-590 can target 3’UTR of the LPL gene and facilitate its mRNA degradation or translational repression, and subsequently inhibit LPL expression. Macrophage LPL can promote development and progression of atherosclerosis by facilitating lipid accumulation and pro-inflammatory cytokine secretion. MiR-590 treatment in apoE^−/−^ mice decreased lipid accumulation and pro-inflammatory cytokine secretion, leading to reduction of atherosclerotic plaque. Therefore, miR-590 has protective effects on atherosclerosis in apoE^−/−^ mice.

In this study, we investigated the atheroprotective effect of miR-590 on atherosclerotic lesions in apoE^−/−^ mice and its underlying molecular mechanisms for miR-590 effects. As summarized in Fig 7, our results revealed that miR-590 inhibits macrophage LPL expression, leading to a reduction in cellular lipid accumulation, the secretion of inflammatory cytokines and atherosclerotic lesions in apoE^−/−^ mice.

Recently, epigenetic factors, especially microRNAs, have emerged as novel components inthe gene expression with regulatory roles in the development of atherosclerosis[26, 27]. MiR-590, a miRNA located on the proximal end of the long arm of human genome chromosome 7, may play a key role in cardiovascular disease via its protection of blood vessels, regulation of lipid metabolism, prevention of myocardial infarction and cardiac regeneration through its target genes[12, 13, 28, 29].In the present study, we observed an inhibitory effect of miR-590 on atherosclerotic lesions in apoE^−/−^ mice with gain-of-function and loss-of-function strategies which were achieved by overexpressing gene products and inhibiting gene expression, respectively[30].

Through tail vein, apoE^−/−^ mice were injected with miR-590 agomir/antagomir or their respective scrambled controls to over-express or inhibit miR-590 expression. Our results showed that miR-590 significantly inhibited the development of aortic atherosclerosis in apoE^−/−^ mice fed high fat/high cholesterol Western diet. Specifically, treatment with miR-590 agomir caused a significant decrease in lesion size and lipid content in aortic wall. Conversely, the atherogenesis was promoted by miR-590 antagomir. To our knowledge, this is the first time to demonstrate that miR-590 has direct protective effect on atherosclerotic progression *in vivo*.

Our studies have also elucidated the potential mechanisms. It is worth noting that increasing evidence suggests that macrophage LPL has the pro-atherogenic feature through stimulating the expression of pro-inflammatory cytokines and lipid accumulation[31–36]. We found that miR-590 directly inhibited LPL protein and mRNA expression by targeting LPL 3’UTR using bioinformatics analyses and dual-luciferase reporter assays*in vitro*[16]. Firstly, the results from the current study showed that CD68-positive macrophages and their LPL expression in atherosclerotic lesions were significantly lower in miR-590 agomir-treated group than its control group. However, miR-590 antagomir treatment enhanced macrophage LPL expression in atherosclerotic lesions. Secondly, miR-590 agomir was found to reduce both LPL mRNA and protein expression in either aortic roots or peritoneal macrophages in apoE^−/−^ mice. Thirdly, macrophage LPL has been identified as a molecular bridge between proteoglycans and lipoprotein receptors, and could promote retention and uptake of atherogenic lipoproteins through up-regulated expression of SR-A or CD36 [25, 37]. As expected, miR-590 agomir treatment reduced both SRA1 and CD36 mRNA and protein expression, whereas miR-590 antagomir treatment increased their expression, when compared with their respective control groups. Taken together, these findings demonstrate an anti-atherosclerotic effect of miR-590 likely through inhibiting the expression of macrophage LPL.

It is known that macrophage LPL has promotive effect on lipid accumulation. Consistently, our data have shown that miR-590 agomir treatment reduced plasma TC and LDL-C levels, but increased TG level. In contrast, miR-590 antagomir treatment increased TC and LDL-C levels, but decreased TG levels in apoE^−/−^ mice, suggesting a role of miR-590 in the regulation of cholesterol levels. LPL is rate-limiting in the provision of triglyceride-rich lipoprotein-derived lipids into tissues[38]. Except macrophage LPL, plasma LPL was also inhibited by miR-590 agomir, resulting in dysfunction of lipolysis and subsequent increase in TG levels. In addition, we measured lipid accumulation in peritoneal macrophages from apoE^−/−^ mice. MiR-590 agomir treatment reduced the levels of TC and CE in peritoneal macrophages of apoE^−/−^ mice, but miR-590 antagomir treatment increased their levels. CE is fairly inert and eventually stored in lipid droplet. During atherogenesis in the presence of some particular factors such as macrophage LPL, oxLDLs may become highly atherogenic as they could induce a significant cholesterol accumulation in subendothelial macrophages, leading to the formation of foam cells[39]. These findings further support that miR-590 may act as a protective factor on atherosclerotic cardiovascular disease.

It has been well accepted that atherosclerosis is a chronic inflammation process[40, 41]. LPL could potentiate proinflammatory cytokine secretion in THP-1 macrophages[35]. In the current study, we have revealed effects of miR-590 on the secretion of inflammation cytokines in apoE^−/−^ mice. Specifically, miR-590 agomir treatment down-regulated the levels of inflammation cytokines, such as TNF-α, MCP-1, IL-1β and IL-6, in both blood and aorta tissues in apoE^−/−^ mice. In contrast, miR-590 antagomir treatment up-regulated the levels of TNF-α, MCP-1, IL-1β and IL-6 in apoE^−/−^ mice. These findings suggested that miR-590 may alleviate inflammation response and subsequently inhibit atherosclerotic progression in mice.

In summary, our novel findings provide evidence supporting that miR-590 is a potential therapeutic target for atherosclerosis in human. However, our understanding of the specific roles of miR-590 in the initiation and progression of atherosclerosis is still limited and at the early stage. Ongoing research should also focus on further development of effective therapeutic methods through targeting miR-590 in treatment of atherosclerotic cardiovascular diseases.

## Supporting Information

S1 File Effects of miR-590 on secretion of plasma pro-inflammatory cytokines in apoE^−/−^ mice. Plasma IL-6 levels in apoE^−/−^ mice as measured by ELISA (Fig A). Plasma IL-1β levels in apoE^−/−^ mice as measured by ELISA(Fig B). Plasma TNF-α levels in apoE^−/−^ mice as measured by ELISA(Fig C). Plasma MCP-1 levels in apoE^−/−^ mice as measured by ELISA(Fig D). Data are shown as the mean±SD. *: P<0.05 *vs*. AG-NC. #: P<0.05 *vs*. AN-NC.
(TIF)
