# Supplementary figures and images for: MicroRNA-590 Inhibits Lipoprotein Lipase Expression and Prevents Atherosclerosis in apoE Knockout Mice
Source: PLoS One. 2015 Sep 23;10(9):e0138788. doi: 10.1371/journal.pone.0138788 (PMC4580638; doi:10.1371/journal.pone.0138788)

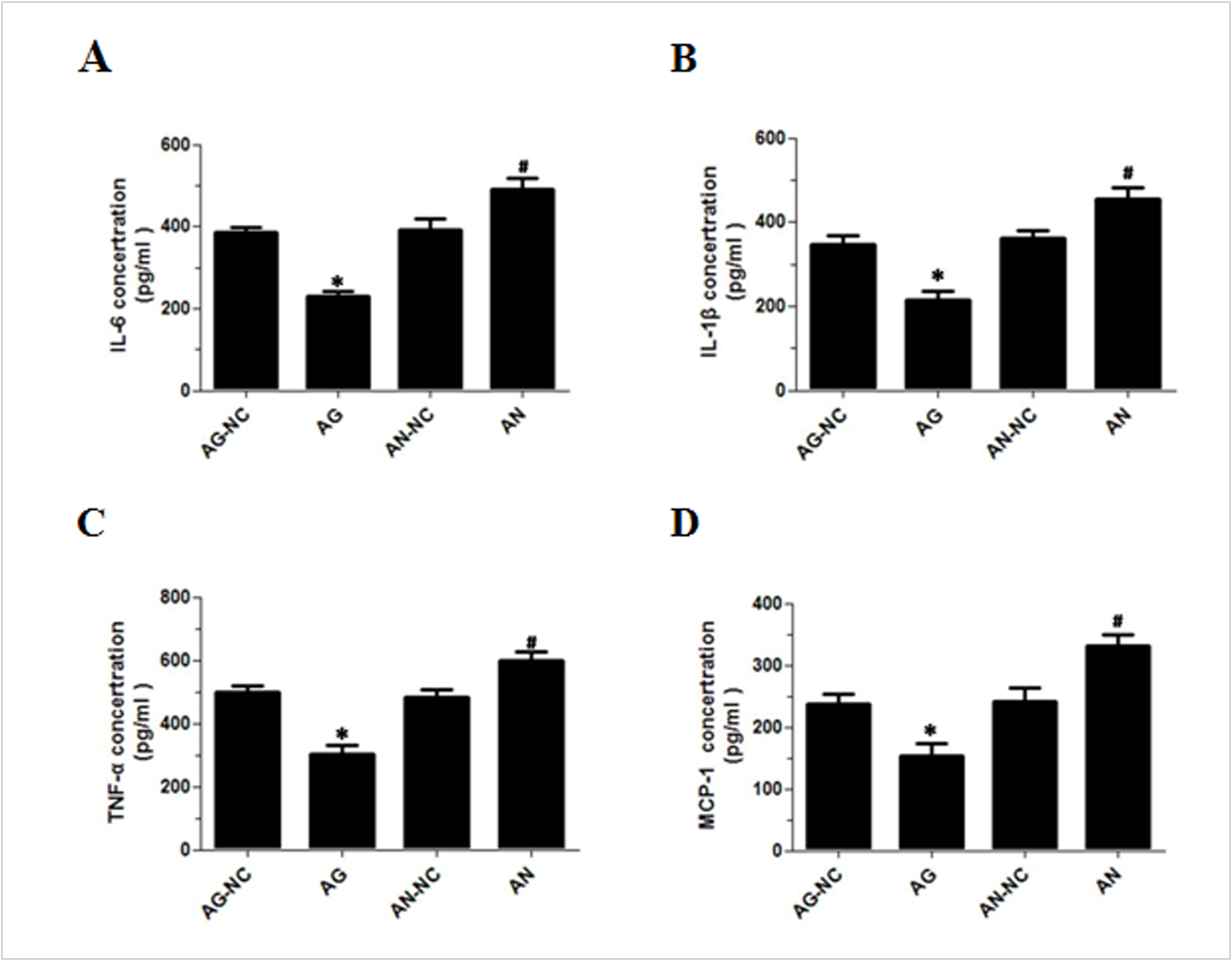

Supplement: S1 File — Plasma IL-6 levels in apoE−/− mice as measured by ELISA (Fig A). Plasma IL-1β levels in apoE−/− mice as measured by ELISA(Fig B). Plasma TNF-α levels in apoE−/− mice as measured by ELISA(Fig C). Plasma MCP-1 levels in apoE−/− mice as measured by ELISA(Fig D). Data are shown as the mean±SD. *: P<0.05 vs. AG-NC. #: P<0.05 vs. AN-NC. (TIF) [file pone.0138788.s001.tif]
